# Describing the Process and Tools Adopted to Cocreate a Smartphone App for Obesity Prevention in Childhood: Mixed Method Study

**DOI:** 10.2196/16165

**Published:** 2020-06-08

**Authors:** Paolo Giorgi Rossi, Francesca Ferrari, Sergio Amarri, Andrea Bassi, Laura Bonvicini, Luca Dall'Aglio, Claudia Della Giustina, Alessandra Fabbri, Anna Maria Ferrari, Elena Ferrari, Marta Fontana, Marco Foracchia, Teresa Gallelli, Giulia Ganugi, Barbara Ilari, Sara Lo Scocco, Gianluca Maestri, Veronica Moretti, Costantino Panza, Mirco Pinotti, Riccardo Prandini, Simone Storani, Maria Elisabeth Street, Marco Tamelli, Hayley Trowbridge, Francesco Venturelli, Alessandro Volta, Anna Maria Davoli, Childhood Obesity Prevention Working Group

**Affiliations:** 1 Servizio Epidemiologia Azienda USL-IRCCS di Reggio Emilia Reggio Emilia Italy; 2 Pediatria Arcispedale Santa Maria Nuova Azienda USL-IRCCS di Reggio Emilia Reggio Emilia Italy; 3 Dipartimento di scienze politiche e sociali Università di Bologna Bologna Italy; 4 Servizio Tecnologie Informatiche e Telematiche Interaziendale Azienda USL-IRCCS di Reggio Emilia Reggio Emilia Italy; 5 Servizio Igiene degli Alimenti e Nutrizione Azienda USL-IRCCS di Reggio Emilia Reggio Emilia Italy; 6 Dipartimento di Salute Pubblica Azienda USL-IRCCS di Reggio Emilia Reggio Emilia Italy; 7 Pediatra di libera scelta, Dipartimento di cure primarie Azienda USL-IRCCS di Reggio Emilia Reggio Emilia Italy; 8 Lepida ScpA Bologna Italy; 9 Medicina dello Sport e Prevenzione Cardiovascolare Azienda USL-IRCCS di Reggio Emilia Reggio Emilia Italy; 10 Dipartimento di cure primarie Azienda USL-IRCCS di Reggio Emilia Reggio Emilia Italy; 11 Servizio di igiene pubblica Azienda USL-IRCCS di Reggio Emilia Reggio Emilia Italy; 12 People's Voice Media Manchester United Kingdom; 13 Clinical and Experimental Medicine PhD Program University of Modena and Reggio Emilia Modena Italy; 14 Cocreation of Service Innovation in Europe Project Reggio Emilia Italy

**Keywords:** childhood obesity, health promotion, mHealth, cocreation, mobile app

## Abstract

**Background:**

Childhood obesity prevention is a public health priority in industrialized countries. The Reggio Emilia Local Health Authority has implemented a program involving primary and secondary prevention as well as the care of obese children. There are many health-promoting mobile apps, but few are targeted to children and very few are sponsored by public health agencies.

**Objective:**

The goal of the research was to describe the process and tools adopted to cocreate a mobile app sponsored by the Reggio Emilia Local Health Authority to be installed in parents’ phones aimed at promoting child health and preventing obesity.

**Methods:**

After stakeholder mapping, a consulting committee including relevant actors, stakeholders, and users was formed. Key persons for childhood obesity prevention were interviewed, focus groups with parents and pediatricians were conducted, and community reporting storytelling was collected. The results of these activities were presented to the consulting committee in order to define the functionalities and contents of the mobile app.

**Results:**

Three key trends emerged from community reporting: being active, playing, and being outdoors; time for oneself, family, and friends; and the pressures of life and work and not having time to be active and socialize. In focus groups, interviews, and labs, mothers showed a positive attitude toward using an app to manage their children's weight, while pediatricians expressed concerns that the app could increase their workload. When these findings were explored by the consulting committee, four key themes were extracted: strong relationships with peers, family members, and the community; access to safe outdoor spaces; children’s need for age-appropriate independence; and professional support should be nonjudgmental and stigma-free. It should be a dialogue that promotes family autonomy. The app functions related to these needs include the following: (1) newsletter with anticipatory guidance, recipes, and vaccination and well-child visit reminders; (2) regional map indicating where physical activity can be done; (3) information on how to manage emergencies (eg, falls, burns, fever); (4) module for reinforcing the counseling intervention conducted by pediatricians for overweight children; and (5) a function to build a balanced daily diet.

**Conclusions:**

The pilot study we conducted showed that cocreation in health promotion is feasible, with the consulting committee being the key co-governance and cocreation tool. The involvement of stakeholders in this committee made it possible to expand the number of persons and institutions actively contributing to the project.

## Introduction

Obesity, its metabolic consequences (eg, hyperglycemia, hypercholesterolemia), and its risk factors (ie, incorrect diet and low physical activity) are responsible for the vast majority of disability-adjusted life years lost worldwide and in industrialized countries in particular [1,2]. In Italy, about 30% of 8-year-old children are overweight or obese [3]. Childhood obesity is one of the major risk factors for adult obesity and diabetes but also has consequences for the child’s health and well-being [4]. This makes childhood obesity prevention a strategic priority in public health, with potential high impact in the medium and long term.

In 2010, the Reggio Emilia Local Health Authority (LHA) started a program of research and interventions aimed at preventing childhood obesity. The program adopted a multilevel and multisetting strategy for primary prevention in the community (particularly in infant-toddler centers and preschools and in primary and secondary schools); secondary prevention (with individual screening for overweight and obese children at age 5 years and counseling by family pediatricians [5,6]); and management of obese children by multidisciplinary teams with treatment of those with complicated pathological obesity. The implementation of such a program increased the need for communication between institutions (LHA, schools, and municipalities), family pediatricians, and families to exchange information about initiatives in school cafeterias, promote physical activity initiatives, facilitate pediatrician counseling, and manage multidisciplinary team activities. New information technologies offer the opportunity to open a bidirectional communication channel between parents and institutions to address these needs.

Recently, several health promotion apps have been developed [7-11], which mostly target adults and adolescents [8,10,12]; very few have been produced by governmental institutions [7-11]. Evaluating the efficacy of these apps as public health interventions is challenging as they often include different functions, making it difficult to separate what works from what does not. Furthermore, although different apps share some of the same or similar functions, the apps are not substantially equivalent to each other in terms of their fundamental components. Thus, systematic reviews cannot pool results from different studies [7]. Some studies are available on apps targeting childhood obesity [13,14], but synthetizing and generalizing their results is difficult.

The efficacy of any health promotion effort depends on its ability to reach and engage the target population. New information technology (IT) tools can help by tailoring the intervention and framing the message according to each family’s needs [15,16]. However, in health promotion and preventive care, the beneficiary’s needs and the health service’s aim often do not correspond. This mismatch may be due to the general public’s lack of awareness of the real impact of diet and physical activity on health but also due to the different value that each individual gives to remaining healthy and changing behaviors. There is, therefore, not only the problem of unmet needs but also of unperceived needs and the fact that a health service and the beneficiary may place different values on prevention.

Cocreation is a process to plan and define public services specifically aimed at reducing the mismatch between beneficiary needs and provided services. Its application in health services and prevention has been recommended [17,18], in particular when IT tools are proposed [19-22]. There are published case studies on the development of apps and other eHealth tools [19,23-30].

The aim of this paper is to describe the process and tools adopted for cocreating an app to be installed on parents’ mobile phones aimed at promoting childhood health and preventing obesity.

## Methods

### Reggio Emilia Pilot Project

This pilot study, using mixed-method research, is one of 9 included in the CoSIE project (Cocreation of Service Innovation in Europe, Call: H2020-SC6-CO-CREATION-2016-2017). This innovative action is aimed at producing guidance on how information and communications technologies can support the process of service cocreation in Europe.

#### Setting

The Province of Reggio Emilia, located in northern Italy, has a resident population of about 530,000 inhabitants, of whom about 80,000 (15.4%) are children ages 0 to 14 years [31]. The province has 6 health districts, one research hospital and 5 district hospitals, and approximately 90 family pediatricians.

#### Bambini Molto in Forma Project

The BMInforma project (Italian: Bambini Molto in forma; English: very fit children) is an ongoing multilevel public health program conducted by the LHA involving primary and secondary childhood obesity prevention interventions. It includes all the LHA’s primary prevention routine activities on childhood health promotion, such as development of school cafeteria menus; extracurricular interventions in infant-toddler centers, preschools, and primary and secondary schools in collaboration with municipal educational services; and collaboration with sports associations to promote organized and nonorganized physical activity. Secondary prevention consists of population-based overweight and obesity screening of children aged 5 years. According to the results of a trial conducted locally [5,6], families of overweight girls are invited to participate in a motivational interview program led by the family pediatrician, while overweight boys receive recommendations and body mass index (BMI) monitoring. Obese children are referred to a multidisciplinary team that organizes group interventions involving family pediatricians, dieticians, and psychologists. Obese children with pathological conditions are referred to the pediatric endocrinology unit at the hospital for care of specific pathologies.

This network of services, initially developed in 2011, is still being fine-tuned. Several research projects are nested in this program, such as a cohort study on distal and proximal determinants of childhood obesity and trials to test the efficacy of individual and group interventions for overweight and obese children. Collaboration with other municipal agencies outside the health sector, such as schools, transportation, and city planning, and with nonprofit organizations is becoming more and more important, in line with the indications of the 2014-2019 National Prevention Plan [32].

**Figure 1:**
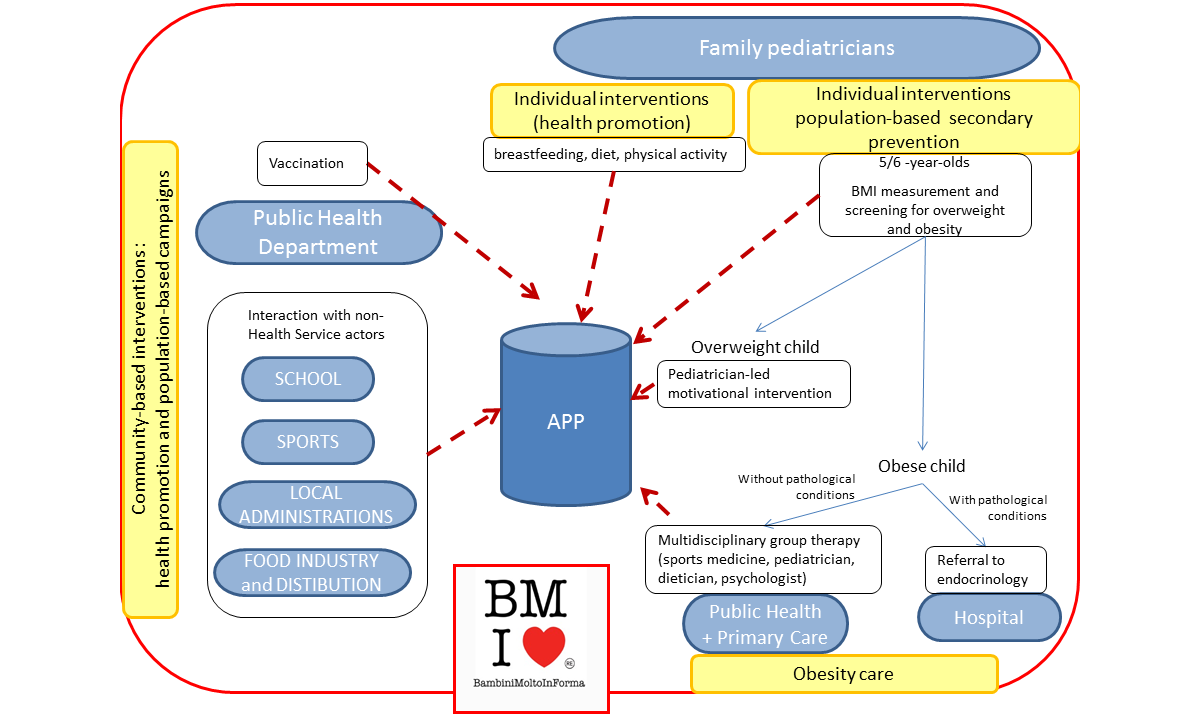
Scheme of the interactions between the primary and secondary prevention and obesity care. The figure also depicts the initial project of an app to improve the service network.

This background makes the BMInforma project a perfect setting to test innovative and traditional tools for cocreating services with families and for co-governance involving the nonprofit and private sectors and the many province-wide municipal administrations (Figure 1).

### Scope of the Needs Assessment Phase

Here we report the results of the needs assessment phase in which we tried to answer the following questions:

- Is the network of initiatives and services on childhood obesity prevention and care meeting the needs of parents and children?
- Are all the components of the network connected and do they share the same objectives?
- How can we improve the network?
- Can an app improve the network?
- What should an app do to be effective?

### Cocreation Tools

#### Stakeholder Map

**Figure 2:**
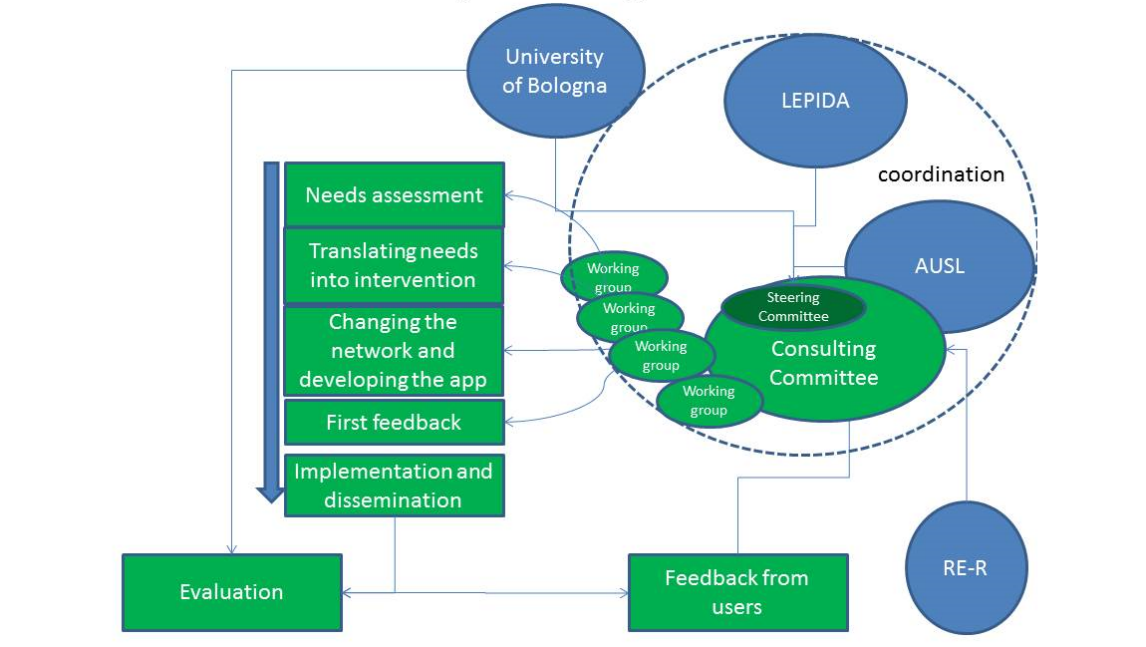
Project management design.

Stakeholder mapping was conducted using an iterative method. Initially, a restricted group of pilot project coordinators, those involved in drafting the application to Horizon 2020, drafted a first list of internal and external stakeholders, decision makers, and beneficiaries of the pilot project. A template was adopted that included the potential influence/contribution of each stakeholder, potential impact of the project on the stakeholder, and possible strategy to involve each stakeholder. Based on this list, a meeting of all internal stakeholders was organized and a new stakeholder analysis was conducted. This step led to the formation of the project steering committee, which included all main internal stakeholders and two experts as external advisors. Finally, the list of stakeholders obtained in the second step was used to create the consulting committee, which included all external and internal stakeholders, decision makers, and parents. During the first meeting of the consulting committee, a third stakeholder analysis was done to identify other public administration sectors and nonprofit associations conducting related projects. The consulting committee decided to remain open to new participants (ie, stakeholders and/or institutions) for the duration of the project and remain active after the end of the project to coordinate local policies on childhood well-being. The project management design, with the main actors and process of evaluation and feedback, is shown in Figure 2.

#### Interviews With Key Actors

The aim of the semistructured interviews was twofold: the first was to evaluate the BMInforma project and changes that the project led and the second focused on a new phase of the project, begun with the Horizon 2020 grant, to determine what had already been done and what was planned on the agenda.

The interviewees, 5 family pediatricians and 3 health care professionals, were identified by the steering committee with the support of the Reggio Emilia LHA. In addition, the 3 project managers of an ongoing trial testing the efficacy of educational group therapy for obese children (Gruppi di Educazione Terapeutica) were interviewed to explore new ways to involve participants (children and their family members) in the pilot research project.

The 8 semistructured interviews were carried out by two researchers from the University of Bologna, Italy, between June and July 2018. Each interview lasted about 1 hour; the same question template was used (see Multimedia Appendix 1 for a detailed outline of the interview).

The main themes to emerge during the interviews:

- Role of family pediatricians and health care professionals in the conduction of both projects (BMInforma and CoSIE)
- Expectations of pediatricians and health care professionals regarding the effectiveness of the app
- The value of cocreation

The semistructured interviews were recorded and transcribed verbatim, and interviewees’ sensitive data were anonymized. Qualitative data analysis was performed using NVivo 12 software (QSR International). A content analysis was then conducted to identify the context where specific nuclei of meaning had been expressed. Codes of meaning, links between all the participants’ statements, conceptual frameworks, and interpretative hypotheses were created using the NVivo software.

#### Focus Groups

Two focus group studies were conducted: one with family pediatricians, the other with parents. The focus group technique, widely used within the social sciences, is an unstructured group interview method that responds to precise rules of preparation, organization, and management. A total of 3 focus group discussions were conducted by the research team from the University of Bologna: one with family pediatricians and two with parents. The mediation of the Reggio Emilia LHA was fundamental in recruiting the participants (both pediatricians and parents). The first focus group discussion was held in May 2018 and involved 14 family pediatricians working in the Province of Reggio Emilia. Thirteen of the pediatricians were female and 1 was male; ages ranged from 30 to 60 years. The focus group discussion lasted about 2 hours (see Multimedia Appendix 2 for a detailed outline of the interview), and the main topics were the role of family pediatricians in dealing with childhood obesity, pediatricians’ relationships with families, and mobile app functions proposed by the consulting committee.

The focus group sessions with the parents (all mothers) were held in October 2018. There were 5 participants in the first and 5 in the second, and ages ranged from 30 to 50 years. Each focus group discussion lasted about 2 hours and the following topics were discussed: lifestyle (how their typical day is organized), nutrition and physical activities, and the role of IT in their lives.

What emerged from the focus groups was analyzed using the same process described for the semistructured interviews, with the addition that word clouds were created through NVivo and used as input during the meetings of the consulting committee.

#### Public Cocreation Lab

During a national festival on digital innovation that took place in Reggio Emilia on October 20, 2018 (during the needs assessment phase of the pilot study), we organized a cocreation laboratory involving families and professionals called “What do you need on your smartphone for your child’s health?” In a family-friendly environment (with organized entertainment for the children), parents had the opportunity to sit down at any one of 4 tables—on diet, physical activity, communication with family pediatricians, or the relationship with municipal institutions—and talk with experts and decision makers about that topic. The contents of conversations with parents were then summarized by the participant experts. There were also other ways to provide input: four signage totems indicating each of the 4 topics were placed around the space, and parents were invited to leave messages, insights, and comments on Post-it Notes. Also, two tablets were placed in the quietest corners of the room for anyone wanting to leave a video and/or audio message. Last, there were whiteboards available to the children and adults for drawing.

To support family participation, Pause and the Reggio Children’s Foundation organized an atelier dedicated to food and tastes in which children could explore vegetables with all 5 senses. All Post-it Notes, notes from the topic table conversations, and videos were then given to the consulting committee without any pre-analysis.

#### Community Reporting

##### Overview

Community Reporting for Storytelling, a pan-European movement established in 2007 by People’s Voice Media, uses digital tools to gather, curate, and mobilize lived experience stories. Its methodological approach is based on the Cynefin decision-making framework for complex environments [33]. Adopting the gathering, curating, and mobilizing community reporting cycle, bespoke interventions across 3 stages were designed and implemented within this study to better understand the needs of families in terms of what keeps them well.

##### Stage 1: Community Reporter Training

Community reporting has three interlinked storytelling models: storytelling, coproduction, and insight. Within this study, the insight approach was used as it provides rich qualitative data to projects by taking the insights from people’s stories to identify a core set of research findings that can be used to inform policy, practice, and service design. Nine participants, including pediatricians, researchers, and members of the pilot project core team, were trained in this approach as part of a 2-day program held May 8-9, 2018, underpinned by peer and experiential learning strategies; the participants explored the following topics:

- The community reporter movement
- Insight storytelling techniques:
  Snapshot stories: short responses to an open question
  Dialogue interviews: unstructured and unscripted interviews with only one preset question used as a conversation starter
- Responsible storytelling and cocreated best practice guide
- Sharing stories online

Using these skills, participants videorecorded a set of stories from families (parents and children) about what keeps them well.

##### Stage 2: Story Gathering and Curation

The community reporters trained in stage 1 gathered more insight stories and uploaded them to the Institute of Community Reporters website [34] from June to September, 2018. The 17 stories gathered during stages 1 and 2 were subsequently analyzed using the Institute of Community Reporters’ analysis model, which examines each story in terms of topic, content, and contextual levels before inductively determining the findings across the stories. In essence, the approach is broadly based on principles associated with established methodologies within discourse analysis [35] and on grounded theory [36].

##### Stage 3: Mobilizing the Insights in the Stories

A conversation of change activity was run October 30, 2018, using findings and extracts from the stories gathered during stages 1 and 2 as part of a consulting committee workshop. Adopting facilitation techniques that drew on aspects of open space technologies, Brené Brown’s vulnerability research and story dialogue techniques [37], stories and findings were used as stimuli for cocreative conversation about what keeps families well.

### Analysis and Synthesis

The consulting committee members received all materials collected in the interviews, focus group sessions, community reporting, and cocreation lab during a plenary session workshop. The materials were organized into the main topics by the social science researchers of University of Bologna and by curators of People’s Voice Media.

The first section of the workshop was a conversation of change activity and involved the findings from the community reporter stories and key exemplary extracts used to prompt thinking about family well-being. Learning from the stimuli was grouped into three categories:

- Key messages of the stories
- Key learning from the stories for health care services
- Experiences of the consulting committee members and how the stories relate

From this, a set of unstructured ideas for the mobile app was produced. This learning was taken forward into the second section of the workshop and combined with other inputs, including focus group sessions, interviews, and the public cocreation lab. Using these, the consulting committee worked in subgroups to summarize and develop the materials in three phases: identifying all possible topics related to family well-being, grouping topics into macro areas that should be covered by the app, and transforming needs into contents or functions the app should include.

**Figure 3:**
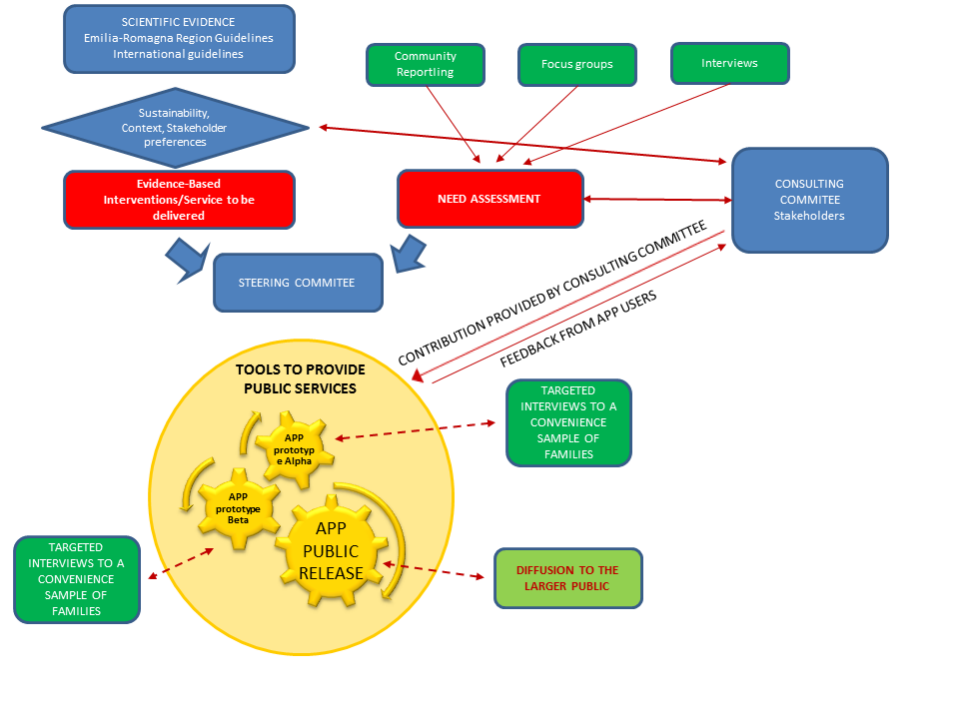
Cocreation strategies for the design, production, and governance of the app in the Reggio Emilia pilot project on childhood obesity prevention.

After the group completed a list of objects/functions the app should have, back-office work was done to produce a list of the app’s requirements to help the technicians of the regional health authority’s IT service produce technical specifications. The subgroups were organized in order to better deal with topics that had similar technical issues. Interventions and services that can be provided to families should be evidence-based and recommended by regional and international guidelines. The choice of interventions to be delivered among those that are evidence-based should be made through a needs assessment phase and an evaluation of sustainability and acceptability in the local context. In this phase, the codesign tools are focus groups, interviews with key actors, and community reporting, all of which are discussed and analyzed by a consulting committee made up of the actors, stakeholders, and users. Synthesis of the input from the needs assessment phase is then conducted by the consulting committee. As soon as different prototypes of the app are delivered, according to the established cocreation approach, interviews with convenience samples of potential target families will be conducted to give feedback and continue the cocreation process. Finally, the app public release will automatically collect feedback from users’ interactions and comments, leading to new iterations of the app (Figure 3).

## Results

### Stakeholder Analysis and Definitions of the Steering and Consulting Committees

The final list of stakeholders included 17 organizations or groups within organizations (Multimedia Appendix 3). Most were in the public sector, but there were also many nonprofit groups and associations and some from the private sector, the latter are mainly food industries and food distribution companies. We decided to involve them through their professional/business associations and not individually, with the exception of one company that currently provides meals to the public schools in Reggio Emilia. Parents participated only through the inclusion of their class representative in the school parent councils. We did not find any parent associations that focused on healthy lifestyle, obesity prevention, or child well-being; the associations we did find focused on abuse prevention, issues related to divorced parents, and the protection of minors. This lack of parent association representative made it extremely important to activate other means of cocreation to receive the users’ and beneficiaries’ inputs.

**Figure 4:**
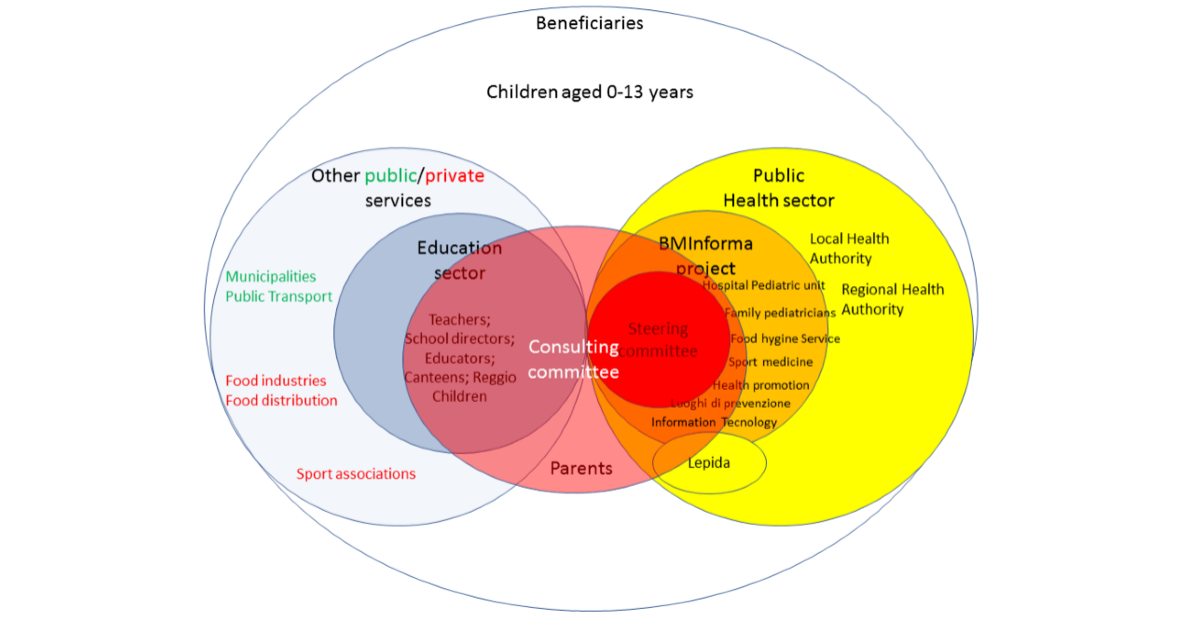
Evolution of the actors and stakeholders and user map.

The process of stakeholder mapping was explicitly designed to increase the engagement of stakeholders who were not initially involved in the project. In fact, starting as the object of the mapping, they became active subjects who defined the map itself throughout the different steps (Figure 4). The process began with the steering committee and the BMInforma project, which was initiated by a group within the LHA. Other departments of the local and regional public health sector were subsequently involved. The establishment of the consulting committee allowed the introduction of other sectors important to children’s well-being, including education, municipal administrations, social innovation, transport, sports associations, the food production and distribution industries, and parent representatives. Furthermore, to increase engagement of the wider community included in the consulting committee, the functions of the steering committee were reduced to preparing the consulting committee meetings, while some of the back-office work (eg, summarizing community reports and the findings of focus groups), initially thought to be the responsibility of the steering committee, was conducted by consulting committee subgroups (Figure 2). The cocreation and co-governance activities led to active involvement in project management by all key actors in the other public, private, and nonprofit sectors on the consulting committee, transforming stakeholders in actors.

### Defining the App Contents: Interviews, Focus Groups, Community Reports, and Cocreation Lab

Interviews provided insight for the evolution of the BMInforma project and the cocreation of the app. All health professionals confirmed the project gave them new skills and more awareness of the problem and sharpened existing skills such as providing appropriate treatments. Observing what emerged from the cocreation of the app, interviewees demonstrated their interest in profiling the app user in order to allow each family to directly assess part of the information in their electronic health file. The personal page might include information such as the school menu and vaccination appointments.

The topics that emerged from the parent and pediatrician focus groups had more to do with the use of IT, and particularly of apps, during their daily activities. Mothers showed great readiness to use an app to manage their child’s weight. In particular, the mothers viewed the possibility of creating new recipes (thus stimulating creativity in the kitchen), checking the family’s diet, and learning about opportunities to improve their habits in a positive manner. The pediatricians raised concerns that the app could increase their workload rather than be an instrument to facilitate work and warned that the app cannot substitute the pediatrician in dealing with the patient’s need.

During the cocreation lab, about 100 Post-it Notes, conversation notes, and 10 drawings were collected. The notes were grouped according to the four topic areas of the lab (diet, physical activity, relationships with public institutions, and communication with pediatricians) to be further analyzed by the consulting committee subgroups. No video or audio messages were collected.

Analysis of the 17 community reporter stories gathered brought to light the following key trends:

- Being active in a variety of ways, often involving play and being outdoors, was seen as important in the families’ lives
- Time to oneself, with family, and with friends was important to supporting the families’ overall well-being
- The pressures of life and work and not having time be be active and socialize were detrimental to families’ overall well-being

Two key anomalies were seen. One story identified how volunteering can support well-being, and another about a young girl’s use of a step-counting bracelet revealed how there can be unintended negative consequences of health (and technological) interventions.

When findings were explored during the conversation of change activity, learning across four key themes was extracted by the consulting committee:

- Strong relationships with peers, family members, and the community support well-being
- Access to safe (outdoor) spaces that can be used for unstructured activities (ie, free play) is important to families
- Children need to have age-appropriate independence to realize and actualize their sense of self and enable them to support their own well-being
- Professional support based on discussion and exchange should be nonjudgmental and stigma-free and promote family independence and their autonomy in making decisions about their lives

### Translating Needs Into App Content

**Figure 5:**
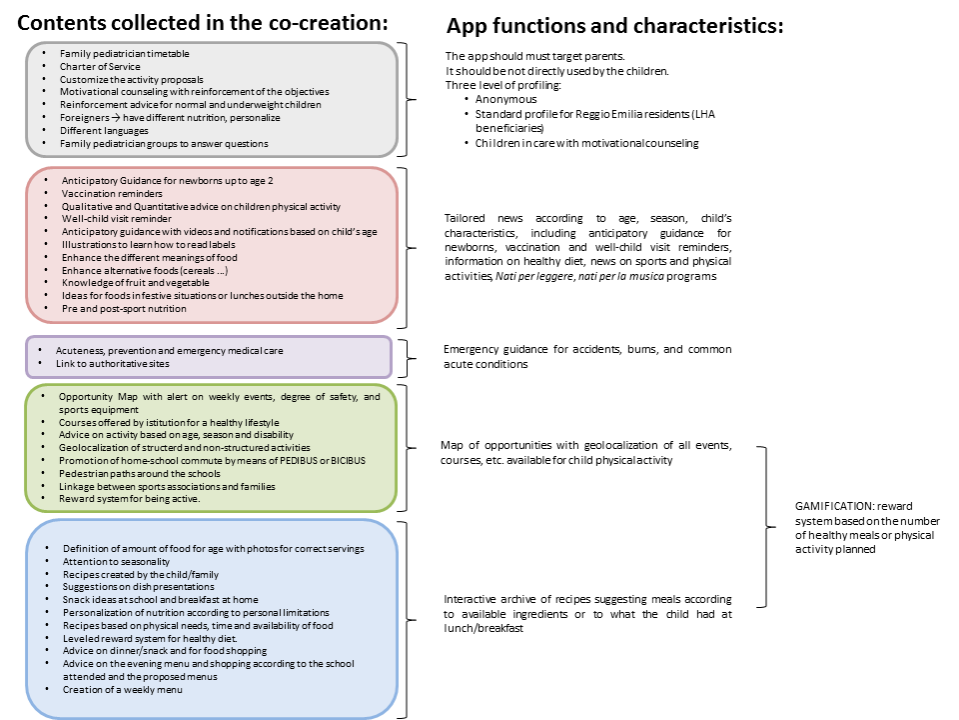
Translating needs into app content.

The consulting committee’s work produced four lists of objects and functions that should be included in the app, divided into main topics: diet and healthy menus, physical activity, relationships with public institutions, and communications with family pediatrician. Several topics were included in more than one list, highlighting the overlap between themes. Small working groups of the consulting committee, integrated with external experts, conducted analyses of the overlapping areas, made observations of technical issues to be addressed, and produced a final list of technical specifications and requirements of the app (Figure 5).

Table 1 compares evidence-based strategies on weight management identified by Rivera et al [38] in a scoping review on mobile apps for weight management with features found in the CoSIE app, which employs 8 of the 11 strategies defined in the review. Only 2 apps (0.5%) in the scoping review included 8 strategies, with an average having between 1 and 2.

**Table 1:** Evidence-based strategies, health care expert involvement, and scientific testing in apps for weight management and in the CoSIE app. Adapted from Rivera et al [38] (n=393).

Evidence-based strategies	Frequency across included apps	CoSIE^a^ app features	
		Present	Comment
Self-monitoring	35.4	Yes	Only for weight
Automatic self-monitoring	10.2	No	Automatic physical activity self-monitoring was not included to avoid causing the children any stress
Goal setting	21.4	Yes	Only for selected users (families with children in obesity prevention program)
Physical activity support	27.5	Yes	In terms of availability of organized and nonorganized physical activities in the community (using a map with activities geo-localized)
Healthy eating support	23.2	Yes	Through recommended recipes in order to have a balanced diet, and information on seasonality of fruits and vegetables and on their nutrients
Weight/health assessment	25.4	Yes	Assessment of body mass index only
Personalized feedback	1.9	No	We exclude personalized feedback for all users because this would involve an unsustainable workload for public health care professionals. However, for children in the obesity prevention program, the app will inform the pediatrician of body mass index monitoring, eating, and physical activity habits
Motivational strategies	7.1	Yes	Through gamification, news, and specific tools for families with children in obesity prevention program
Social support	5.3	No	We exclude online communication with other users since it would involve supervision and control, which we cannot guarantee at the moment
Health care expert involvement in development	0.3	Yes	—
Scientific test	0.8	Yes	In progress

^a^CoSIE: Cocreation of Service Innovation in Europe.

## Discussion

### Principal Findings

The cocreation process described here succeeded in producing a list of the content of the childhood obesity prevention app; the content items proposed by the different cocreation process participants and from the users’ (parents) and beneficiaries’ (children) suggestions were largely consistent. Mixing several cocreation tools, some of which were more conventional (eg, forming a consulting committee) while others were more innovative (eg, community reporting and public laboratories), made it possible to balance input from citizens and institutions. Finally, the process succeeded in transforming the consulting committee into an active community that brought together different sectors, favoring synergy and operating in a “health in all policies” perspective [39].

### Strengths and Limitations

This study is merely descriptive; we cannot rule out that similar or even better results and a similar level of decision sharing could be reached with another process. Furthermore, we have reported the process used to determine app content; we do not yet have any information about how the app will actually be used and whether it will be effective in promoting healthy behaviors in children.

A critical point was parent involvement in the consulting committee. The core group proposed not to include specific parent associations because of their focus on some aspects of parenting; we initially included only a representative of the school parent councils, a choice supported by the other stakeholders on the committee. The limited representation of final users on the consulting committee resulted in an orientation more toward co-governance than cocreation. In the literature, in fact, co-governance does not involve any of the beneficiaries; co-governance refers to the joint participation only of public agencies, the private sector, and nonprofit organizations in decision making and the planning of public services [40-42]. The parents’ suggestions had to be collected with more unconventional cocreation tools (ie, focus groups, community reporting, and public lab), as commonly described in similar experiences [17,43-47]. Nevertheless, as the target of our initiative on obesity prevention is the families themselves, many of the stakeholders on the consulting committee are parents or grandparents of children in the target age (from newborn to preadolescence) and thus also potential end users, and many of them have also faced the issue of overweight/obesity in childhood, given that the prevalence is close to 30%.

The fact that many members of the consulting committee were simultaneously stakeholders and potential users made our cocreation process different from all the previous experiences of cocreation in health interventions (including apps and eHealth products), where end users provided input on the needs and tested the prototypes but were not involved in the identification of the aims or in the conduction of the project [43-47]. In our pilot project, the process of cocreation through the consulting committee activities gradually eliminated the borders between the core pilot project leader group and the other stakeholders and end users involved. This process became clearer when the tasks originally assigned to the steering committee (ie, synthesizing the needs assessment phase into a document suitable for technical requirements and content of the app) were transferred to working groups made up of consulting committee participants.

### Topics That Emerged During the Needs Assessment Phase

We obtained a detailed list of content and requirements suitable for technical development of the app. Adopting strategies proposed in a previous review on weight management apps, the CoSIE app as outlined by the cocreation process fulfills most of the quality requirements (Table 1) [38].

Surprisingly, content and topics families proposed were consistent with those identified by the institutions, with a few exceptions. For example, during the public lab, a couple of parents asked for an online pediatrician to provide quick answers to questions posted through the app; the health care professionals, however, considered this unfeasible. In the scientific literature, we found only one study in which parents could directly contact a dietician or psychologist through the app [48,49]. Although the authors did not report any issues regarding health practitioner workload induced by the app-mediated contacts, the study was conducted on a small sample and scaling up to the whole target population of this intervention was not proposed because it was not effective in reducing fat mass index and changing behaviors [49]. Further, LHA officials expected a request to simplify bureaucracy through the app, but no beneficiary made this request. The main theme emerging from parent input was the need for time and for safe public spaces. This was the main concern of most of the institutions as well, who look for ways to increase physical activity. The shared emphasis was on how to facilitate access to playgrounds and other community spaces for children that are suitable for safe, unstructured play. The app’s map of opportunities, which lists all the places in a given neighborhood where it is possible to play and be physically active, could respond to this request; such a function was not common in other similar apps [50]. Lack of time was a key issue in parents’ requests regarding the promotion of a healthy diet and a positive relationship with food. In this case, the only tool an app can offer is the cookbook, with two ways to access it: according to the ingredients in your refrigerator or to your preferences. Some apps evaluated in scientific reports [15,16,48,49] allow self-monitoring of food consumption and weight status, with automatic feedback on correct energy uptake. While providing feedback on daily or weekly diet is still under consideration in the CoSIE app within the gamification and rewarding function (although not considered a high priority by the consulting committee), any direct contact with a health care professional has been expressly ruled out. In general, stakeholders proposed avoiding any prescriptive approach. The CoSIE app does not propose any diets, physical activity programs, or tutorials, unlike similar apps [15], because behavioral changes should come about thanks to a favorable environment and attitude.

What to do in case of an emergency was another topic both parents and health practitioners requested. This topic is not strictly related to obesity prevention and, to our knowledge, not present in other similar apps, but it was recognized as a way to make the app useful and appreciated by parents.

A proposal for gamification of the app emerged particularly from the institutions, who saw it as a way to engage families. Both users and institutions agreed that the app should not be used directly by the child but by the parent only (ie, it is on the parent’s smartphone). This is the main barrier to gamification but also guarantees that any game is played with a parent or at least under the parent’s supervision. The solution was to develop games only for designing a healthy menu and planning the right physical activity over the course of a week.

### Cocreation in Health Services: Insights From the Pilot Study

In general, health services have several specific characteristics that make cocreation particularly challenging. Analyses have both highlighted the theoretical benefits of involvement strategies to health care (eg, promoting equity and improvement) and identified numerous tensions and contradictions that play out in practice [17,23,51-54]. Nevertheless, within this project, particular emphasis was placed on improving each phase [55] of the process by harnessing the experiences of experts, citizens, and patients. Here we described the first two phases, co-commissioning and co-design, but we have already planned the next steps of co-delivery and co-assessment of the entire obesity prevention program, not just of the app.

The main difference between public health services and other public services is that the former demand scientific evidence of the efficacy of any intervention before it can be provided. In the case of primary prevention, we often see interventions with scientifically proven efficacy, but they have rarely been compared in head-to-head experiments measuring the relative efficacy of different interventions with the same aim. Furthermore, in most cases different preventive interventions are not mutually exclusive, and using interventions together can be a successful strategy [56]. Therefore, the national health system should decide which interventions are better suited to a given context, more acceptable to a given population, sustainable given the available resources, and thus recommended by the National Prevention Plan [32,57]. Having a short list of recommended interventions limits the opportunity to implement a thorough cocreation process. On the other hand, the need to apply some criteria to prioritize interventions and adapt the best intervention for a given community among those recommended is a process that can obtain enormous benefits from the application of a cocreation process.

Prevention and health promotion interventions raise another issue: these interventions, particularly those aimed at changing risky behaviors and promoting healthy lifestyles, are not perceived as needs by the target population [58,59]. The target population often considers these interventions unwelcome intrusions in their lives [60]. Furthermore, in prevention, the target population may attribute a value to the possible health benefits of an intervention that is substantially different from that attributed by society (or by public health care professionals). In our specific case (ie, childhood obesity), we observed in our previous studies [5] that many parents were not at all aware that their child was overweight, and that in some cases, parents’ main concern was underweight, even when their child was frankly obese. Again, cocreation tools (in this study, community reporting and focus groups) involving parents who became aware that their children were overweight and health care professionals with experience in counseling these families gave us valuable insight into what is actually perceived as the most important issues in improving the family lifestyles.

When the service provided is a prevention intervention targeting childhood obesity, the definition of users and beneficiaries is also challenging; while the beneficiaries are the children themselves, we are interested in changing the entire family’s behaviors (diet and physical activity are both determined by family habits). Finally, in our pilot project, the users of the app will be only the parents; a positive effect on the beneficiaries (children) can only be achieved by changing the behaviors of the larger target (the family) [61,62].

### Mobile Apps in Health Care Service: Cocreation of a Cocreation Tool

The next step of the process will be the development of the app prototype during which conversations with the consulting committee subgroups will be still in place. The first app prototype will be tested by users (families and pediatricians). Initially, user feedback will be collected through focus groups. The app itself will collect qualitative and quantitative feedback; the use of different services in the app will be logged and it will be possible to comment on the various app functions by means of a short text or simply clicking on a like or do not like icon.

Cocreation in health promotion and prevention has some unique features that must be taken into consideration. The pilot study we conducted showed that cocreation is feasible. The key co-governance and cocreation tool was the consulting committee. Including stakeholders on this committee made it possible to expand the number of persons and institutions actively contributing to the project.

## Appendix

Multimedia Appendix 1 Interview outline.

Multimedia Appendix 2 Focus group outline.

Multimedia Appendix 3 Stakeholder matrix.

## Abbreviations

BMI: body mass index
BMInforma: Bambini Molto in Forma
CoSIE: Cocreation of Service Innovation in Europe
IT: information technology
LHA: local health authority
