# Supplementary material for: Describing the Process and Tools Adopted to Cocreate a Smartphone App for Obesity Prevention in Childhood: Mixed Method Study
Source: JMIR Mhealth Uhealth. 2020 Jun 8;8(6):e16165. doi: 10.2196/16165 (PMC7308901; doi:10.2196/16165)
Supplement: Multimedia Appendix 1 [file mhealth_v8i6e16165_app1.docx]

COSIE PROJECT

REGGIO EMILIA - PILOT

INTERVIEW OUTLINE

A] – Short bio

(Education- professional training, job experience, current occupation and role)

B] – Breakdown into two subcategories, B1 in reference to CoSIE, B2 with respect to the BMI project.

B1 Role in COSIE - motivations, expectations, workload

(How did you hear about the project? Who involved you in the project? What is your role in Cosie? Why did you join? What are your expectations for the project? What led you to joining? What do you think your workload on the project will be? Do you receive any form of compensation for your participation?)

B2 BMI Project

(How and when did the BMI project start? What exactly does it consist of? What was the basic idea or philosophy? What was your role in activating the BMI project? What functions do you perform in the project? Are you satisfied with the results achieved so far? How do you see the project 3 years from now? What are the main effects of the BMI project on the population?)

C] – Activities carried out to date - progress of the project [COSIE]

(Have you participated in any activities so far? If so, what is your opinion on the matter? Degree of satisfaction, etc. What did not work? What should be done, in your opinion?)

D] – Expectations - impacts - temporal dimension [COSIE]

(What do you expect to get from your participation in CoSIE? What do you think should be done to avoid undesired effects or ineffectiveness? What impacts do you think CoSIE can have on your work setting?).

E] – Value of Co-creation

(Do you think that health is an area where citizens can be included to co-produce health services? What do you think could be the potential benefits of co-creation in health services? And the limits? How do you build conditions that foster co-production of health services? Can involving citizens / patients in treatment planning benefit therapy?)

F] Culture of well-being and lifestyles

Can you tell us what your opinion is on the following topics:

1) whether institutions can legitimately dictate the standards of healthy lifestyles;

2) the capacity of the institutions involved in this obesity prevention project to have a real impact on citizens' health.

3) the relationship between various cultures of well-being (i.e. those who come from the Southern Italy, or from other countries) and that promoted by the institutions;

4) what the main obstacles are to spreading an institutional culture of well-being.
