# Supplementary material for: Describing the Process and Tools Adopted to Cocreate a Smartphone App for Obesity Prevention in Childhood: Mixed Method Study
Source: JMIR Mhealth Uhealth. 2020 Jun 8;8(6):e16165. doi: 10.2196/16165 (PMC7308901; doi:10.2196/16165)
Supplement: Multimedia Appendix 2 [file mhealth_v8i6e16165_app2.docx]

**Focus group outline**

Four thematic areas have been identified that could be addressed during the focus group:

a) lifestyle (eating habits, physical activity, relationships, leisure time);

b) the influence of the setting (environment)

c) technology and health (app)

**a) Lifestyle**

1. Introductions (who they are and what they do).
2. What is your typical day like?
3. Could you define the main problems / challenges you encounter in your day in "managing" your children?
4. How would you define your eating habits? Who usually prepares meals at home?
5. Can you briefly describe how you and your family get around?

[e.g., means of transportation to go to work? What role do the grandparents play?]

1. Can you tell us about your relationship with the neighborhood you live in? [are there parks, bicycle paths? Do you use them? In what moments / periods / occasions?]
2. What do you like doing in your free time?

**b) The influence of the setting (environment)**

1. What is your opinion the school canteen? What are the main negative aspects and what could be done to solve them?
2. Do you think the level of physical activity practiced during school hours is satisfactory?
3. Do your children play sports outside school hours? [if NO, why not?]
4. Do you consider sports and physical activity, organized (courses and activities organized and scheduled by sport clubs and societies) and non-organized (for example playgrounds, public green areas) accessible?
5. Do you know of any initiatives promoted by the Municipality/volunteer associations regarding health promotion for children, in particular healthy diet and physical activity?

**d) Technology and health (app)**

1. Does your family use any electronic devices (smartphone, PC, tablet)?
2. Do your children have their own smartphone/tablet/PC/etc.? How often do they use them to play games/watch cartoons? If not, do they use your device(s)?
3. Have you ever used an app (for smartphone, tablet, PC) to monitor your own health or that of a relative?
4. Would you use an app to monitor daily nutrition?
5. Would you use an app to prevent obesity?
6. What do you think would make that app effective? What features do you find most useful?
