# Supplementary material for: Describing the Process and Tools Adopted to Cocreate a Smartphone App for Obesity Prevention in Childhood: Mixed Method Study
Source: JMIR Mhealth Uhealth. 2020 Jun 8;8(6):e16165. doi: 10.2196/16165 (PMC7308901; doi:10.2196/16165)
Supplement: Multimedia Appendix 3 [file mhealth_v8i6e16165_app3.docx]

| **Stakeholder** | **Geographical coverage** | **Impact**  *How much does the project impact them?* | **Influence**  *How much influence do they have over the project?* | **Stakeholder’s key interests** | **How could the stakeholder contribute to the project?** | **How could the stakeholder block the project?** | **Strategy for engaging the stakeholder** |
| --- | --- | --- | --- | --- | --- | --- | --- |
| *Primary care department of the Local Health Authority* | Local | High  (actor) | High  (internal) | To coordinate the primary care activities (including FP and General Practitioners (GP)). To conduct the negotiations with FP and with GP unions for incentives and additional activity fees | Organizing outpatient care for obese children.  Making specific agreements with FP. | Low prioritization. Lack of dedicated resources. | The stakeholder is part of the project; there is no need to engage further. |
| *Family Pediatricians (*FP*)* | Local | High  (actors) | High  (FP coordinators: internal; other FPs: external) | To care for beneficiary children. To promote healthy lifestyle. To conduct motivational interviews. To screen for obese children. | Conducting the interventions. | Low commitment. | Coordinators are very involved.  Strategies for involving all the FPs should be agreed on. |
| *Communication Service of the Local Health Authority* | Local | High  (actor) | High  (internal) | Relations within the Local Health Authority and with citizens and other external entities. | Coordinating project activities with other local Health authority activities. Implement communication strategies for dissemination (website, etc.) | Low commitment, lack of dedicated resources. | Provide regular updates on the project activities and results. |
| *Pediatric Unit –Santa Maria Nuova Hospital* | Local | Medium  (actors) | Medium  (internal) | Care of complicated obese children.  Research prevention and treatment effectiveness | Interface for II and III level. Conduct research on second level intervention (“GET study” is ongoing) | Low commitment.  Not taking care of referred patients.  Caring for children that should be referred to less intensive setting. | Establishing a Steering Committee.  Clear protocols defining eligibility criteria for II and III level. |
| *Hygiene, Food and Nutrition Service of the Local Health Authority*  *(SIAN)* | Local | High  (actors) | High  (internal) | Assessment and consultation of food delivery in schools and dietary habits. Healthy diet promotion. | Monitoring food delivery for children and promoting policies on healthy food. | Low dissemination of COSIE activities and results. | Establishing a Steering Committee.  Integrating SIAN activities into the core of the project (APP) |
| *Public Health and Hygiene Services of the Local Health Authority*  *(*including health promotion and sport medicine*)* | Local | Medium  (actors) | High  (internal) | Promotion of lifestyle. Sports-related health issues and certificates. | Coordination of health promotion activities (including link with Luoghi di Prevenzione)  “Map of physical activity opportunities in the province”. | Lack of coordination with other health-promoting activities.  The “Map” is not updated or unsuitable for APP dissemination. | Establishing a Steering Committee.  Integrating the project activities into a plan of school- and community-based health-promoting activities.  Integrating the “Map” into the APP |
| *Information and Communication Technologies Service of the Local Health Authority* | Local | High  (actors) | High  (internal) | Security, privacy, and integrity of the data;  Use of Information and Communication Technology (ICT) standards. | Supporting the implementation of the ICT tool. | The ICT tool is not fully integrated with the IT systems. | The stakeholder is part of the project; there is no need for involvement |
| *Nurseries (0-3 yrs); preschools (3-5 yrs); elementary schools (6-11 yrs); middle schools (11-13 yrs)* | Local/ regional/ national | Medium  (actors) | High  (external) | Promotion of healthy lifestyle. | Advocacy for families and help them learn about the importance of diet and physical activity. | Low commitment and poor participation. | Different forms of participation in the Consulting Committee. |
| *Regional Health Authority* | Regional | Low  (actors) | High  (external) | Promoting best practices | Advocacy, Policy development and future scale-up of the project at regional and national level | No endorsement.  Low compliance with the standards used in the regional eHealth network | Checking consistency between regional guidelines, standards, and proposed interventions. Establishing a Consulting Committee. |
| *Parents* | Local/National | High  (beneficiaries) | High  (external) | Engagement of families in the co-creation process. | Foster motivation in the participating families. | Low dissemination and poor involvement. | Establishing a Consulting Committee. |
| *Joint Consultative Committees (*including volunteer association representatives+members of the Local Health Authority+Representatives of General Practitioners and municipalities*)* | Local | Low  (beneficiaries) | Low  (external) | Promoting the quality of the healthcare services and equity in access. Guarantee citizen participation in healthcare system decisions and presents the citizen needs. | Give voice to citizens in the co-creation approach. | Not sharing the same vision of project activities. | Establishing a Consulting Committee. |
| *Reggio Children* | International | Low  (actors) | Medium  (external) | Research, study, and development aimed at high-quality education for all. | Support in dissemination and communication. | No endorsement. | Establishing a Consulting Committee. |
| *Luoghi di prevenzione* | National | High  (actors) | High  (internal) | Identifying innovative actions for health promotion and prevention of diseases. | Include the COSIE activities in their programs and projects. | Not sharing the project vision. Not caring for referred children. | Clear protocols defining eligibility criteria for individual and group therapy. Including their activities in the APP. |
| *Committee of Foreigners* | Regional | Low  (beneficiaries) | Low  (external) | Inclusion of disadvantaged groups. | Give voice to citizens in the co-creation approach for immigrants. Currently, this committee is not very active. |  | Establishing a Consulting Committee. |
| *Municipalities*  *of Reggio Emilia Province* | Local | Medium  (actors) | Medium  (external) | Administering the municipality, providing non-health-related services to citizens (preschool, transportation, social services, public green spaces, playgrounds, city planning, etc.). | Building an environment that fosters healthy choices. | No endorsement. Making policies not favoring the project. | Establishing a Consulting Committee. |
| *Food industry, catering agencies, and food distribution* | Local | Low (actors) | Low (external) |  | They should apply recommendations to school menus and food distribution. They also sponsor some health-promoting activities not directly related to the project, which must be coordinated | Non-compliance with recommendations about menus and food distribution. | To be developed with food hygiene service |
| *Sports associations* | National, Local | Low (actors) | Low (external) |  | They participate in the definition of the map of physical activity opportunities. They also participate in some health-promoting activities. | Not collaborating in defining the map of opportunities | To be developed with sports medicine |

**Note: for some stakeholders, different levels or ways of involvement are proposed.*
